# Correction: Lee et al. Improved Optical Efficiency of 850-nm Infrared Light-Emitting Diode with Reflective Transparent Structure. *Micromachines* 2023, *14*, 1586

**DOI:** 10.3390/mi15020241

**Published:** 2024-02-05

**Authors:** Hyung-Joo Lee, Jin-Young Park, Lee-Ku Kwac, Jongsu Lee

**Affiliations:** 1 CF Technical Division, AUK Corporation, Iksan 54630, Republic of Korea; dieblood77@nate.com; 2 Korea Photonic Technology Institute (KOPTI), Gwangju 61007, Republic of Korea; police7630@kopti.re.kr; 3 Department of Carbon Convergence Engineering, Jeonju University, Jeonju 55069, Republic of Korea; 4 Department of Advanced Components and Materials Engineering, Sunchon National University, Suncheon 57922, Republic of Korea

In the original publication [1], the acknowledgment “This work was supported by the National Research Foundation of Korea (NRF) grant funded by the Korea government (MSIT) (No. 2021R1C1C1013387)” was not included in Funding section. The authors state that the scientific conclusions are unaffected. This correction was approved by the Academic Editor. The original publication has also been updated.

## References

[1] Lee H.-J., Park J.-Y., Kwac L.-K., Lee J. (2023). Improved Optical Efficiency of 850-nm Infrared Light-Emitting Diode with Reflective Transparent Structure. Micromachines.

